# Optical Coherence Tomography Is a Promising Tool for Zebrafish-Based Research—A Review

**DOI:** 10.3390/bioengineering10010005

**Published:** 2022-12-20

**Authors:** Antonia Lichtenegger, Bernhard Baumann, Yoshiaki Yasuno

**Affiliations:** 1 Center for Medical Physics and Biomedical Engineering, Medical University of Vienna, 1090 Vienna, Austria; 2 Computational Optics Group, University of Tsukuba, Tsukuba 305-8573, Japan

**Keywords:** zebrafish, optical coherence tomography, angiography, polarization-sensitive, multi-modal imaging pre-clinical, biomedical research

## Abstract

The zebrafish is an established vertebrae model in the field of biomedical research. With its small size, rapid maturation time and semi-transparency at early development stages, it has proven to be an important animal model, especially for high-throughput studies. Three-dimensional, high-resolution, non-destructive and label-free imaging techniques are perfectly suited to investigate these animals over various development stages. Optical coherence tomography (OCT) is an interferometric-based optical imaging technique that has revolutionized the diagnostic possibilities in the field of ophthalmology and has proven to be a powerful tool for many microscopic applications. Recently, OCT found its way into state-of-the-art zebrafish-based research. This review article gives an overview and a discussion of the relevant literature and an outlook for this emerging field.

## 1. Introduction

The zebrafish (*Danio rerio*) is an established and important vertebrae animal model in modern biomedical research [1]. Since its first introduction as an animal model by Streisinger et al. in the early 1980s [2], this fish, which measures up to 35 mm, has found its way into many research fields, such as pre-clinical cancer studies, toxicology and precision medicine [3,4,5]. 

**Figure 1:**
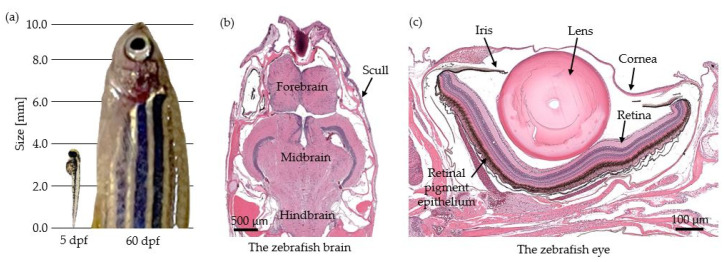
The size of the zebrafish and selected histology images. (**a**) Size comparison between 5 and 60 days post-fertilization (dpf). (**b**) Hematoxylin and Eosin (H&E)-stained transverse histology section of the zebrafish brain. (Link to the Bio-Atlas slide: http://bio-atlas.psu.edu/zf/view.php?s=193&atlas=16, accessed on 1 October 2022 [8]). (**c**) H&E-stained histology image of the zebrafish eye. (Link to the Bio-Atlas slide: http://bio-atlas.psu.edu/zf/view.php?s=182&atlas=16, accessed on 1 October 2022 [8]). Both histology images were retrieved from a one-year-old wildtype animal.

There are multiple advantages of utilizing zebrafish over traditionally used rodent models such as mice. A female fish can lay up to 200–300 eggs per week, leading to a fast and relatively cheap breeding scheme. Moreover, the rapid maturation time, their small size and the low housing costs have paved the way for this animal model in low-cost and high-throughput investigations [1,3]. After three days post-fertilization (dpf), the embryonic phase is already completed and is followed by a 6 week period of larval development. The juvenile fish reaches sexual maturity within three months and is then considered to be an adult [6]. Figure 1a shows a size comparison of a larva at 5 dpf and a juvenile fish at 60 dpf, highlighting the rapid development of this animal model. Even though it is not a mammal, humans and zebrafish share a high degree of genome structure, as around 70% of human genes have at least one obvious ortholog in zebrafish [7].

Each animal model also possesses shortcomings; the zebrafish is a poikilothermic animal which lives in the water, and therefore some physiological processes and organ structures differ from those in mammals. Nevertheless, research has shown that most tumors formed in these animals resemble human ones [9,10]. Furthermore, over the past decades, advanced genetic modification techniques have been established, enabling forward and reverse genetic analysis, thus leading to the possibility of disease monitoring in an in vivo organism and advances in drug discovery screenings [11]. In comparison to murine models, the semi-transparency of the zebrafish larvae makes live imaging possible using conventional microscopic techniques [7]. Furthermore, zebrafish larvae lack an active immune system during the first 12–14 days of their development, facilitating some investigations, for example, in xenograft studies where immune suppression would be needed to be able to transplant tissue from a different species into the animals [12].

**Figure 2:**
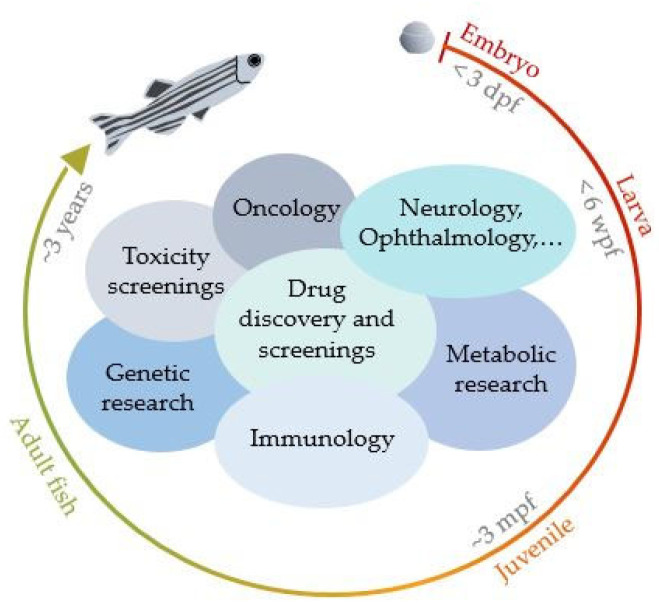
Biomedical applications/research areas in which zebrafish models have been utilized. (Days post-fertilization—dpf, weeks-post-fertilization—wpf, months-post-fertilization—mpf).

Figure 2 gives an overview of various biomedical applications and research areas in which zebrafish models have been used. The development stage of the zebrafish needs to be chosen according to the investigated disease, organ and type of study [1,7].

Zebrafish-based research performed at early development stages by means of white light or fluorescence microscopic techniques takes advantage of the fast maturation time, small size of the animals and the semi-transparency. These imaging techniques offer cellular resolution and are compatible with markers to enable tissue specific contrast [13]. One important type of fluorescence imaging used in zebrafish investigations is light sheet fluorescence microscopy (LSFM) [14,15]. This technique enables cellular resolution and imaging of thicker tissue slices with reduced photobleaching and phototoxicity compared to standard fluorescence microcopy. However, to generate LSFM image contrast, some type of fluorescent molecule must be incorporated within the tissue. Additionally, specific manipulations of the sample are needed to create a semi-transparent sample, i.e., removing or clearing pigments and other optically opaque substances [16]. Confocal microscopy (CM) has been widely utilized to study zebrafish, such as in brain or cardiovascular investigations. CM can visualize cellular features in real-time in embryos and larvae [17,18]. Furthermore, time-lapse imaging is a powerful and non-invasive approach to examine blood vessels and perform heart rate monitoring in early zebrafish development stages [19].

Another promising label-free imaging technique to investigate zebrafish at early development stages is Mueller-matrix imaging. Using the polarization information, tissue specific contrast is gained which is of interest when analyzing changes in early musculature development. However, this technique is typically limited to early larval stages due to light scattering and absorption [20,21].

For some investigations, for example, in pre-clinical cancer research, it may be beneficial to examine juvenile or even adult animals [22]. Due to light absorption and scattering, the imaging range in advanced development stages is limited to sub-dermal structures. The work of White et al. showed that one possibility to overcome this limitation is to develop genetically-modified, semi-transparent zebrafish strains, which lack specific skin pigmentations, to facilitate the imaging process [23]. However, this procedure introduces additional working steps and more complicated breeding schemes [23,24].

Alternatively, a variety of other imaging techniques have been utilized to investigate juvenile/adult zebrafish models, such as micro magnetic resonance imaging (MRI), computed tomography (CT) and other tomographic techniques, such as holographic and photoacoustic imaging [25,26,27,28]. MRI and CT have several limitations, such as the complexity, high cost involved and the potential hazard caused by the high magnetic field or radiation [25,26]. Photoacoustic imaging has proven to be a promising research tool, although the resolution is typically limited to a couple of ten to hundred micrometers [27]. It would be beneficial to establish a compact and low-cost imaging modality which can examine non-transparent zebrafish models in 3D over various development stages with a micrometer, high-resolution capability in a non-destructive way.

Optical coherence tomography (OCT) is an imaging technique based on low-coherence light interferometry which was introduced in the 1990s. The technique was originally developed to investigate the eye and has proven to be a game changer in the field of ophthalmology [29]. Over the past decades, OCT has found its way into many other biomedical research fields, such as dermatology, endoscopy and neurology [30,31,32]. Furthermore, microscopic applications, for example, in vitro cell spheroid, ex vivo tissue or in vivo small animal investigations, revealed novel insights [33,34,35]. Table 1 highlights the capabilities and drawbacks of the most common imaging technologies used for in vivo zebrafish-based research.

To generate an OCT signal, a light beam is sent to the sample, which is reflected and backscattered by the internal structures of the tissue. By interfering this reflected and backscattered light with a reference beam, interference fringes are generated and are collected by a detector. Through data post-processing, a depth profile of the sample, or A-scan, is generated. By scanning the beam across the sample, a cross-sectional tomogram, or B-scan, and a volumetric representation are gained. OCT is a real-time technique where 3D OCT volumes can be acquired in a couple of seconds [29]. The bandwidth of the light source defines the axial or depth resolution. The lower the central wavelength and the broader the spectrum, the higher the resolution becomes, which typically is in the range of 1–15 μm [36,37]. The lateral resolution is defined by the imaging optics. One defines an OCT system as an optical coherence microscope (OCM) if an objective lens with a high numerical aperture (NA) is used to focus the light beam onto the sample. The available depth range is a combination of the used optics and spectral range. As a rule of thumb, the lower the central wavelength and the higher the resolution, the shorter the imaging range [37,38]. Please find additional information about the conventional terms used in OCT research in the supplementary material (Figure S1 and Table S1).

Over the last decades, new contrast modalities for OCT were developed. The most relevant so far in the field of zebrafish-based OCT research are elucidated in this review article. Polarization-sensitive OCT (PS-OCT) is a functional extension of conventional intensity-based OCT. By analyzing the polarization states of the backscattered light, the polarization properties of the tissue can be measured and used as an additional source of contrast [39,40]. The polarization of light describes the spatial orientation of the electromagnetic waves. Two polarization parameters, which can be measured by PS-OCT, are birefringence and depolarization [39,40]. Birefringence is a tissue property commonly found in highly orientated structures such as fibrous tissues. In such materials, light with different polarization components experiences different refractive indices and hence, propagates in different speeds in the sample. The difference between the refractive indices of the two eigenpolarizations, i.e., fast and slow polarization components, is called birefringence and can be measured by a PS-OCT setup [41,42]. Polarization scrambling or depolarization occurs when the polarization state of a light beam experiences a random change while traveling through a material. In PS-OCT imaging, the so-called degree of polarization uniformity (DOPU) has been frequently used as a parameter to characterize depolarization [43]. Other depolarization metrics which have been introduced include the degree of polarization (DOP) or the entropy [44,45]. Many research groups have shown that PS-OCT can add additional contrast information and can therefore improve the assessment of various tissue types and diagnostic processes [46,47,48].

Another groundbreaking development in the field of OCT was the introduction of OCT angiography (OCTA). OCTA can visualize the vasculature in a label-free way [49]. This technique is based on the fact that static and moving particles can be differentiated by taking repeated cross-sectional images at precisely the same location and analyzing the intensity or amplitude differences of the backscattered OCT signal. OCTA contrast can therefore be gained by a standard OCT setup by adapting the acquisition and the data processing. Vasculature changes are an important indicator for many types of diseases, making OCTA examinations a good candidate for identifying new biomarkers to detect early disease onsets [50,51].

This review article highlights the progress and importance of OCT-based zebrafish research. First, an analysis was performed to show the distribution of the included research articles thematically. In the following chapters, the presented articles are grouped into related topics and are introduced. The discussion section gives a critical view on the literature so far and an outlook for the future.

## 2. Literature Review

### 2.1. Categorical Literature Analysis

A keyword-based publication search was performed using the defined keywords: “Optical coherence tomography AND zebrafish”, “Optical coherence microscopy AND zebrafish”, “OCT AND zebrafish” and “OCM and zebrafish” in PubMed and Scopus, including all articles until the 1st of December 2022. After excluding all non-English articles, duplicates were removed, and for the remaining 60 included articles, a full-text screening was performed. The data evaluations and illustrations were performed in Excel (Microsoft Office).

**Figure 3:**
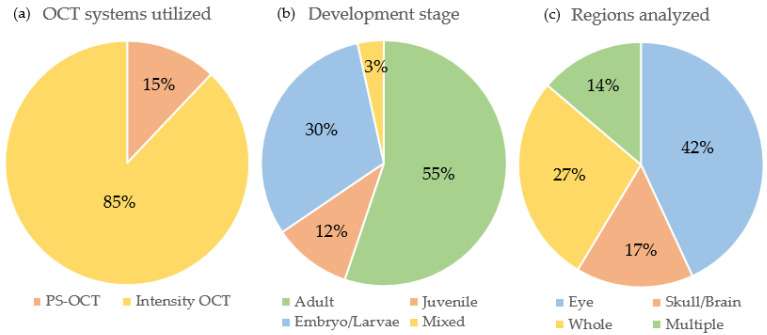
Categorical analysis of the included articles. (**a**) Type of optical coherence tomography (OCT) setups used (Intensity-based OCT setups (Intensity OCT), Polarization-sensitive OCT (PS-OCT)). (**b**) Investigated development stages. (**c**) Anatomical locations or regions examined.

First, an analysis was performed to identify various categories; the results are shown in Figure 3a–c. This analysis showed that 85% of the studies included in this review were performed using intensity-based OCT setups; however, some had additional contrast channels such as fluorescence, OCTA or photoacoustic imaging, and 15% were utilizing polarization-sensitive OCT (see Figure 3a). When analyzing the investigated development stage of the zebrafish, 55% of the research groups used adult, 12% used juvenile, 30% used embryos/larvae and 3% used a variety of multiple age groups in their studies (see Figure 3b). Finally, we analyzed the anatomical locations investigated by the different research articles. Nearly half of the studies (42%) were conducted in the fish eye, 17% in the head/brain region, 27% investigated the whole fish body and 14% examined multiple, distinct anatomical locations in their studies (see Figure 3c).

### 2.2. Investigating Zebrafish Embryos and Larvae

**Figure 4:**
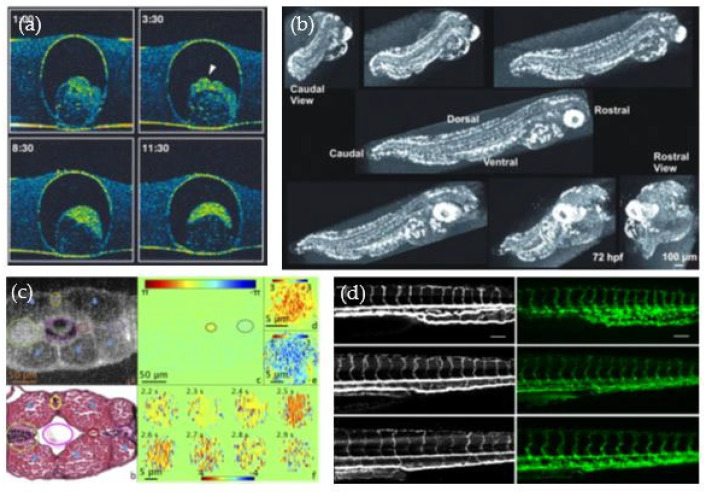
OCT studies performed at early development stages. (**a**) The embryonic development imaged with OCT [52]. Re-print permission was granted by the Creative Commons Attribution license (CC BY 4.0) by Cambridge University Press. (**b**) OCT imaging of a zebrafish larvae [54]. Re-print permission was granted by the authors and Mol. Vis. Journal. (**c**) Doppler OCT reveals blood flow in the trunk of zebrafish larvae quantitatively. Reprinted with permission from Haindl et al. [55] ^©^ The Optical Society. (**d**) OCT angiography was used to investigate the blood vessels in the tail of a zebrafish larva [56]. Re-print permission was granted under the Creative Commons (CC BY 3.0)—Gold Open Access by SPIE.

The first work in the field of OCT-based zebrafish research was published in 1996 by Boppart et al. to investigate the embryonic development of the zebrafish. The early embryonic development in the fish egg was examined using a 1300 nm OCT setup with an axial resolution of 16 μm and a transverse resolution of 30 μm (see Figure 4a) [52]. These results were also included in a review article about OCT for developmental biology a couple years later [53].

The group of Kagemann et al. used an OCT setup with an axial resolution of 3.4 μm to investigate multiple developing structures such as the eye, the brain and the yolk sac in zebrafish from 24 h-post-fertilization (hpf) up to 120 hpf (see Figure 4b). Additionally, using repeated line scanning dynamic processes, such as the heartbeat, were examined [54]. To achieve an axial resolution of 1.0 μm, Cui et al. combined two super luminescent diode sources and showed cellular details in zebrafish larvae [57]. To be able to image the zebrafish larvae with an even higher axial and lateral resolution over a large imaging depth, Chen et al. moved to a lower central wavelength, a broad spectrum covering 760–930 nm and a quasi-Bessel beam illumination [58]. Recently, Zhou et al. introduced 3D optical coherence refraction tomography, a computational extension of OCT to create speckle-reduced and refraction-corrected volumes of 2 dpf old zebrafish larvae [59]. Furthermore, the investigation of ethanol-induced developmental defects in zebrafish embryos showed that OCT is a favorable tool for a non-invasive assessment of birth defects in small animal systems [60].

Many disease types are associated with an initial abnormal growth of the vasculature [61]. That is why various pre-clinical studies of zebrafish development were focused on investigating perfused blood vessels using different techniques, such as phase variance OCT, Doppler OCT and OCTA [59,60,62,63,64]. Among others, Doppler OCT enables quantitative blood flow measurements, showing, for example, that the maximum flow in the ventral aorta (11-μm in diameter) in 5 to 8 dpf old zebrafish is 32.4 mm/s [63]. The results demonstrated that OCT can visualize and quantify the vasculature in a non-invasive and label-free way, i.e., no contrast agent is needed (see Figure 4c,d).

### 2.3. Studies Performed in the Zebrafish Eye

Originally, OCT was developed to study the human eye in a non-invasive way. Similarly, most studies (42%) performed in zebrafish-based research so far were also focused on the eye (see Figure 3c). The zebrafish eye shows a similar anatomical hierarchy compared to the human eye, including a cornea, a lens and a retina (see Figure 1b), and is therefore an attractive model to study various eye development processes and diseases [65].

Most of these studies were conducted in adult fish using intensity-based OCT setups to image the retina [66,67,68,69,70,71,72,73,74,75,76,77,78,79,80,81,82,83,84,85,86,87]. Primarily, retinal investigations were performed using commercially available OCT setups, such as the Leica (former Bioptigen) Envisu R2200 [68,69].

So far, only two studies have been performed for the anterior zebrafish eye. One study utilized a spectral-domain OCT (SD-OCT) setup to investigate the regenerative potential of the zebrafish corneal endothelium by evaluating the thickness of the cornea after a surgically induced injury [88]. SD-OCT is a common type of OCT setup in which a spectrometer is used as a detector to analyze the generated interference signal (please see the supplementary material for a more detailed explanation) [29]. The other study used a SD-OCT setup to measure the axial eye length and other dimensions as a tool to characterize myopia and emmetropia in zebrafish [89].

**Figure 5:**
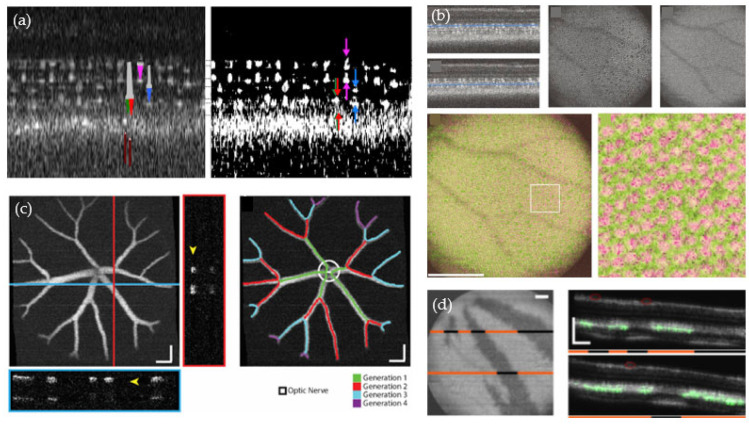
OCT studies conducted in the zebrafish retina. (**a**) High-resolution OCT imaging of the fish eye reveals the microscopic features of the retina [85]. Re-print permission was granted by the Creative Commons Attribution license (CC BY 4.0) by Cambridge University Press. (**b**) The cone mosaic visualized using OCT [86]. Re-print permission was granted by the Creative Commons Attribution license (CC BY 4.0) by Cambridge University Press. (**c**) Quantitative biometry of the zebrafish retinal vasculature. Reprinted with permission from Bozic et al. [73] ^©^ The Optical Society. (**d**) Visualizing the melanin distribution in the zebrafish retina using photothermal OCT [71]. Re-print permission was granted under the CC BY-NC-ND license by ARVO.

Various OCT studies were focused on assessing the zebrafish retina, the effects of retinal damage and the regeneration process [66,67,77,78,79,81,82,83,84,85]. Figure 5a shows microscopic anatomical features of the retina imaged with a high-resolution OCT setup [85]. The cone photoreceptors in the retina are a fundamental anatomical structure for vision. Thus, several OCT studies investigated the density of the cone mosaic before and after ablation and regeneration [68,69,86,87]. Zebrafish possess various types of photoreceptors, including the ones for ultraviolet (UV) and the ones for red/green vision. A false-color cone mosaic image of these two types of photoreceptors obtained with an 878-nm commercial Bioptigen Envisu R2200 SD-OCT system is shown in Figure 5b. The retinal pigment epithelium (RPE) is another structure in the back of the eye that has been examined using OCT [70,71,72]. Lapierre-Landry et al. used a photothermal OCT system to visualize the melanin distribution in the zebrafish retina (see Figure 5d). They modified a commercial OCT system (Envisu R-2200, with a central wavelength of 860 nm) by adding an excitation laser diode for the photothermal signal, revealing melanin-specific information. The technique differentiated pigmented and non-pigmented as well as dark and light-adopted wildtype zebrafish based on the photothermal signal [71].

Since many diseases of the eye are associated with changes of the vasculature, some effort has been made to characterize and quantify the retinal vasculature in healthy and diseased zebrafish models, such as in acute retinal hemorrhage, using OCTA [73,74,75,76]. Bozic et al. introduced a longitudinal OCTA analysis tool to perform quantitative vascular biometry which could support researchers working on large-scale zebrafish eye studies (see Figure 5c) [71]. Recently, Quint et al. presented an in vivo screening model to study ocular phenotypes of zebrafish using OCT [80]. One can conclude that investigations in zebrafish models for ophthalmology, in combination with OCT, is a thriving research field to examine the anatomical features of the fish eye, as well as to identify disease relevant markers.

### 2.4. Studies Performed in the Zebrafish Skull and Brain

**Figure 6:**
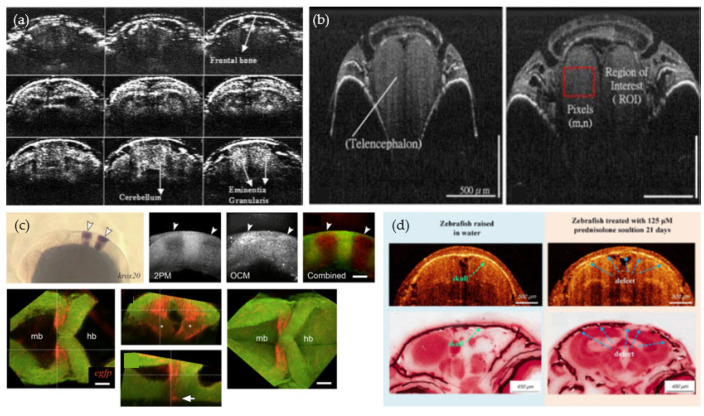
OCT studies performed in the zebrafish skull and brain. (**a**) In vivo brain imaging at 1310-nm [92]. Copyright Wiley-VCH GmbH. Reproduced with permission. (**b**) Investigating the aging process of the zebrafish brain using OCT [95]. Copyright Wiley-VCH GmbH. Reproduced with permission. (**c**) Embryonic brain development examined with a multimodal OCT system [96]. Re-print permission was granted under the Creative Commons (CC BY 3.0)—Gold Open Access by SPIE. (**d**) Osteoporotic process examine using OCT. Reprinted with permission from Lin et al. [97] ^©^ The Optical Society.

The zebrafish brain shows many anatomical similarities and homologies with the human brain, such as the presence of a fore, mid and hindbrain (see Figure 1c). As such, the zebrafish is an attractive model organism to study various neurological and neurodegenerative diseases [90]. The zebrafish brain in adult animals is around 2 mm at its thickest position. It is protected by a bony skull and covered by connective tissue and skin which is in total around 200 μm thick [65]. Wang et al. showed that by using an OCT setup operating in the near infrared wavelength region (1300 nm), a depth range up to 2.5 mm in human brain tissue can be investigated [91]. The ability to widely cover the zebrafish brain and the capacity for high-resolution imaging makes OCT a great tool to investigate this organ in a non-destructive and label-free way. In 2009, Rao et al. introduced a swept source OCT (SS-OCT) setup, which is another common type of OCT (see supplementary material) operating at 1310 nm to perform real-time in vivo brain imaging in adult animals [92] (see Figure 6a). In 2015, Zhang et al. published their work on in vivo adult zebrafish brain imaging using a SD-OCT system with a light source at a central wavelength of 1325 nm and a video-rate imaging speed of 27 frames per second. Results in animals with puncture wounds in the brain region as well as control wildtype were presented, suggesting that OCT can be used as a tool to investigate wound healing processes [93]. Utilizing the injection of amyloid-beta protein, the group of Lin et al. investigated long-term brain atrophy using OCT. Histology analysis confirmed the atrophy as well as the presence of amyloid-beta protein in the zebrafish brain. Interestingly, they also reported a memory loss, assessed through behavior tests, associated with the accumulation of the amyloid-beta protein. Their research shows the potential of OCT for neurological research, such as in the field of Alzheimer’s disease [94].

In 2019, the same group utilized a light source at 846 nm to achieve an axial resolution of 7.2 μm, with a custom-built SD-OCT system, to investigate the osteoporotic process in the adult zebrafish skull (see Figure 6d). For this investigation, wildtype animals at 90 dpf were used and the osteoporotic process was introduced through a chemical component called prednisolone. OCT imaging was performed over 21 days following induction. Using the OCT results, bone defects were identified over time and the results showed a good agreement to conventional histology [97]. Recently, the group investigated the regeneration process of the skull using a chemical compound called phycocyanin. They used three different groups of fish: without treatment and with low and high doses of the compound. After creating a skull defect, all animals were imaged over a period of 20 days with their SD-OCT system at 830 nm. Their results showed that OCT can be utilized to evaluate the right concentration of phycocyanin for cranial defect recovery [98].

Lin et al. used a commercial OCT setup (SR-OCT, OCP930SR, Thorlabs, Newton, NJ, USA) in combination with signal attenuation evaluation to identify the age of the fish brain (see Figure 6b). The standard length (body size) and age in larvae and juvenile zebrafish is linearly related; however, after 132 dpf the growth rate declines. In the presented study, the authors investigated 20 animals older than 90 dpf, in a size range of 1 cm up to 3.5 cm in body length, and showed that OCT signal attenuation can be used as a potential technique to examine and monitor the age of the zebrafish brain and its development [95].

Furthermore, OCM in combination with two-photon microscopy (2PM) has been utilized to examine the embryonic brain development (see Figure 6c). The OCM contrast provided a visualization of the brain morphology and the 2PM added the desired molecular contrast [96,99].

### 2.5. Novel Contrast Mechanisms

There is a constant development in the field of OCT, including identifying promising new mechanisms for improved tissue contrast, either based on advanced OCT technology such as PS-OCT, magnetomotive OCT or by fusing OCT with other imaging techniques, such as photoacoustic imaging.

**Figure 7:**
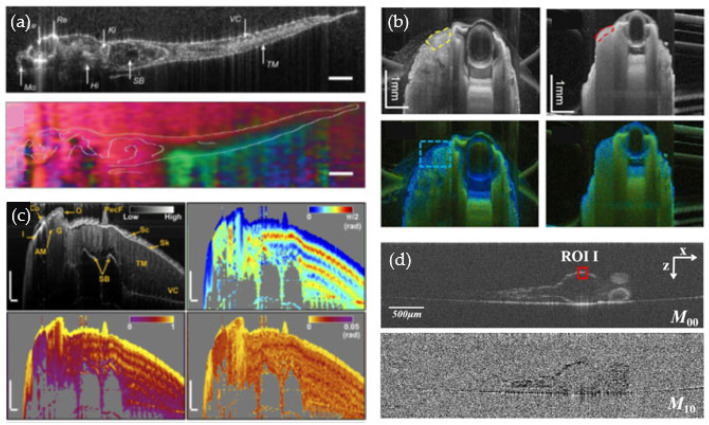
Polarization-sensitive OCT (PS-OCT) studies. (**a**) First PS-OCT studies in postmortem zebrafish [100]. Reprinted with permission from Palmieri et al. [100]. (**b**) Zebrafish model showing a tumor next to the eye. Reprinted with permission from Lichtenegger et al. [104] ^©^ The Optical Society. (**c**) Muscle investigation in wildtype animals. Reprinted with permission from Yang et al. [108] ^©^ The Optical Society. (**d**) Yolk sac evaluation using PS-OCT [107]. Copyright Wiley-VCH GmbH. Reproduced with permission.

Recently, PS-OCT setups have been utilized for zebrafish-based research. First, in 2015, Rossignoli et al. performed post-mortem PS-OCT imaging of 30 dpf old zebrafish using a light source centered at 1550 nm [100]. Their results showed that structures such as the muscles can be investigated with an improved contrast provided by the polarization information [100] (see Figure 7a). Yang et al. demonstrated the usage of a PS-OCT setup at a central wavelength of 840 nm to study the zebrafish musculature (Figure 7c) and the skin [101,102]. Using this PS-OCT setup, the group recently presented impressive results for brain and skull imaging of six-month old zebrafish. They showed that the polarization information can be used to image animals whose skulls have been removed, and monitor the recovery process of the head wound [103]. Jones-matrix OCT (JM-OCT) is a specific sub-type of PS-OCT which can measure the depth-resolved birefringence by a Jones-matrix analysis [41]. Using such a setup, Lichtenegger et al. first examined wildtype zebrafish over three development stages, namely 8 dpf, 1 and 2 months post-fertilization, as well as postmortem adult tumor models [35,104]. The polarization-contrast enabled an improved identification of tumor regions close to the eye (see Figure 7b) and in the zebrafish brain. Based on these results, Lichtenegger et al. further investigated a juvenile xenograft tumor model in vivo using the same JM-OCT prototype to show the feasibility of a long-term study to identify tumor related changes over time in the tail musculature [105]. To further improve the additional image contrast gained by the JM-OCT polarization-sensitive data, Zhu et al. introduced a computational refocusing strategy which mitigates induced polarization artifacts, such as incorrectly high birefringence values [106]. Li et al. used a type of PS-OCT setup to image and segment the yolk sac in control and malformed animals [107] (see Figure 7d). The articles published so far emphasized that PS-OCT can add additional tissue-specific contrast to improve the characterization of anatomical features, such as the muscles or the spine, and can even enhance the identification of tumor regions [35,100,101,102,104,107].

Multi-modal OCT, combining OCT and other microscopy methods have been introduced for zebrafish-based research in order to expand imaging capabilities. The group of Liu et al. examined zebrafish by combining photoacoustic microscopy (PAM) and OCT [108,109,110]. In photoacoustic tomography, the sample is illuminated with short laser pulses; chromophores in the tissue absorb this energy and convert it into heat and acoustic waves. The photoacoustic waves are then detected via a piezoelectric transducer which generates the PAM image contrast. Photoacoustic imaging provides deep tissue imaging, especially for blood vessel investigations. In their publication, the authors demonstrated that PAM/OCT (OCT axial and lateral resolutions: 8 μm and 20 μm, PAM axial and lateral resolution: 34 μm and 88 μm) can image the inner organ dynamics even in adult zebrafish [109]. The next generation of their combined OCT (axial and lateral resolution 5 μm and 3 μm, respectively) and PAM (axial and lateral resolution 286 and 4 μm, respectively) system enabled the investigation of finer details in the morphology of zebrafish larvae [110]. The most recent iteration of combined OCT and PAM provided an even better resolution capability (OCT axial and lateral resolution: 3.7 μm and 3.4 μm, PAM axial and lateral resolution: 37.3 μm and 1.9 μm) based on a fiber optic sensor, demonstrating the potential of multimodal setups for investigations of the anatomy and the vasculature of zebrafish larvae [101].

Kim et al. integrated a 663 nm diode pump laser to enable tissue-specific imaging by using methylene blue with their 830 nm OCT setup. Methylene blue has a variety of clinical applications and is, for example, a common stain used in chromoendoscopy of the gastrointestinal tract. By using the pump laser and their advanced post processing algorithm, they showed an accumulation of methylene blue in specific zebrafish organs [111].

Furthermore, the development of Janus microspheres as a positive contrast agent showed improved OCT contrast possibilities [112]. The group fabricated Janus microspheres from polystyrene and half coated them with gold to increase the back reflection and scattering intensity for OCT imaging. These approximately 2 μm diameter spheres were then orally administrated to adult wildtype zebrafish, resulting in improved OCT image contrast in the stomach region.

Recently, magnetomotive OCT (MM-OCT) has been used for non-invasive and three-dimensional tracking of magnetic nanoparticles (MNPs) and has been applied to zebrafish-based research [113]. To generate the MM-OCT contrast, an electromagnet modulates a magnetic field during imaging, changing the orientation of the MNPs which were injected into the zebrafish hypodermis. This change in orientation of the MNPs within the skin region can be observed as a shift in the OCT signal, leading to a tissue-specific contrast.

## 3. Discussion

Since the first publication of OCT imaging of embryonic zebrafish development in 1996 [52], many research groups have used OCT technology to investigate this unique animal model. As highlighted in this review article, numerous studies have explored OCT for eye imaging in zebrafish, as well as for various other fields, such as neurology [92], developmental studies [54], wound healing [98,102] and oncology [104].

In comparison to imaging techniques, such as MRI or CT, OCT provides a smaller imaging depth but is less cost intensive, does not rely on potentially hazardous radiation or high magnetic fields and is more compact, making it easily accessible for the lab environment [25,26]. An additional strength of OCT as a non-invasive and label-free imaging technique is its speed, which provides real-time capability, i.e., the tissue morphology can be examined “live” during the scan [29]. However, one limitation of conventional intensity-based OCT setups is the lack of molecular contrast. Research groups around the world have been focusing on solutions to overcome this drawback. One possibility is the combination of OCT with complementary techniques such as fluorescence imaging, which labels specific molecules while OCT gives access to the 3D tissue morphology [96,99]. As demonstrated in these publications [96,99], this combination may be a powerful tool in future zebrafish-based research.

Furthermore, recent results demonstrated by polarization-sensitive OCT have shown that this approach could add the desired tissue specific contrast in a label-free way [35,100,101,102,104,107]. In particular, the specific identification of fibrous structures through a birefringence analysis showed an improved identification of muscle groups, skin lesions and the diagnosis of tumor regions [35,101,102,104]. Alternatively, motion contrast mechanisms based on advanced OCT signal analysis have been introduced, such as OCTA [50,51] or Doppler OCT [114,115]. OCTA is already an established method for disease diagnosis in the field of ophthalmology for human eyes [50]. For zebrafish-based research, OCTA has proven capable of analyzing and quantifying the vasculature in a label-free way and investigating disease types such as acute retinal hemorrhage [59,60,62,63,64,73,74,75,76]. Dynamic OCT has revealed fascinating insights into ex vivo and in vitro samples [116]. In a first attempt to perform dynamic-OCT in vivo, a full-field OCT setup was utilized to image a zebrafish larvae, revealing additional cellular features [117]. However, to adapt this technique for in vivo measurements at later development stages, the challenge will be to overcome the required long acquisition times, for example, by increasing the imaging speed [118].

**Figure 8:**
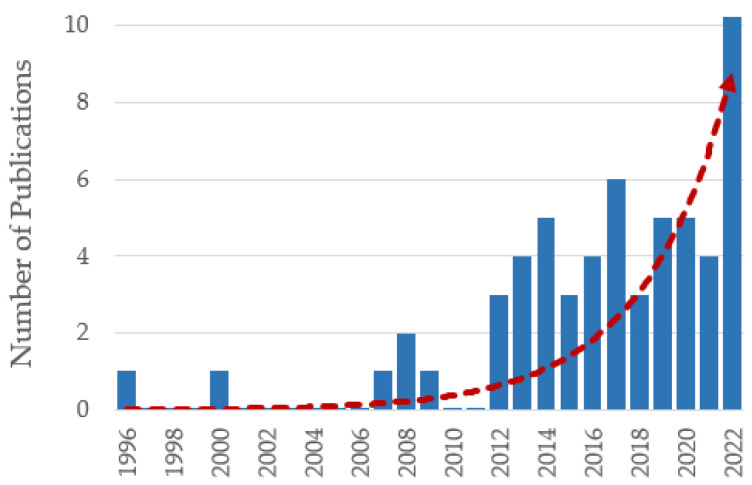
Number of publications in OCT-based zebrafish research over the last decades. The data from the categorical literature analysis were utilized to create this graph. The red dashed line indicates the exponential trend line.

OCT has revolutionized the diagnostic possibilities in the field of ophthalmology and has found its way into other application areas, such as dermatology, endoscopy and neurology [30,31,32]. So far, most OCT setups have been manufactured for human eye diagnosis; however, more and more companies and research groups are working towards OCT systems for pre-clinical studies and clinical translation [119]. The authors believe that this trend will allow researchers working with zebrafish-models from around the world to explore OCT for their scientific questions, which is reflected in the increasing number of articles published since 1996 (see Figure 8).

For the future, there are multiple other OCT-based technologies which have not yet been used for zebrafish imaging, such as OCT elastography [120]. New technological developments in the field of OCT are pushing towards sub-cellular resolution using OCM, which will enable the identification of even finer anatomical features competing with conventional microscopy [121]. In general, in OCT, there is a tradeoff between resolution and imaging depth, depending on the used wavelength region. The optimal choice for zebrafish-based research strongly depends on the development stage investigated, and therefore the body size and the specific research question. Furthermore, we believe that one important step to establish OCT in this field is the refinement of setup specifications for zebrafish-based research. For example, automated well plate screening with OCT could better enable high-throughput studies [9].

The studies presented in this review article already covered a variety of topics. In the future, OCT might be applied in other fields to evaluate fast toxicity screenings, especially for the inner organs of adult animals [122], or in extended drug application studies, tumor investigations [123] and precision medicine [124]. For example, in the field of precision medicine, OCT could support the evaluation of the zebrafish as a human avatar to test possible treatment options in a label-free, real-time and non-destructive way.

While OCT is on its way to becoming an established technology in the field of zebrafish-based research, many exciting results have already been achieved, as shown by the wide range of applications presented in this review.

## Figures and Tables

**Table 1:** Comparison of various imaging technologies and OCT used for in vivo zebrafish imaging. The resolution capability, the possible depth range, the contrast mechanism, if the technique is label-free and the costs involved are compared.

Imaging Technique	Resolution	Depth Range	Contrast	Label-Free	Costs
Positron emission tomography	mm-range	Whole adult fish	Radiation	No	High
Magnetic resonance imaging	μm/mm-range	Whole adult fish	Magnetic field	Yes	High
Computed tomography	μm/mm-range	Whole adult fish	Radiation	Yes	High
Photoacoustic imaging	10–100 μm	mm-range	Light/photoacoustic waves	Yes	High/Moderate low
Optical coherence tomography	1–10 μm	mm-range	Light	Yes	Moderate low
Fluorescence microscopy techniques	>200 nm	Hundreds of μm in unprocessed samples	Light	No	Low/ Moderate low
White-light microscopy	>200 nm	Surface imaging	Light	No	Low

## Data Availability

Not applicable.

## Supplementary Materials

The following supporting information can be downloaded at: https://www.mdpi.com/article/10.3390/bioengineering10010005/s1, Figure S1: Different types of optical coherence tomography (OCT) setups; Table S1: Comparison of system parameters of selected publications in the field of OCT-based zebrafish re-search. References [29,52,54,55,59,60,64,68,73,80,93,98,103,105,107,125,126,127,128] are mentioned in the supplementary materials
