# Supplementary material for: Optical Coherence Tomography Is a Promising Tool for Zebrafish-Based Research—A Review
Source: Bioengineering (Basel). 2022 Dec 20;10(1):5. doi: 10.3390/bioengineering10010005 (PMC9854701; doi:10.3390/bioengineering10010005)
Supplement: Supplementary file 1 [file bioengineering-10-00005-s001.zip › bioengineering-2052447-supplementary.pdf]

bioengineering

MDPI

Review

Optical Coherence Tomography Is a Promising Tool for Zebrafish-Based Research—A Review

Antonia Lichtenegger, Bernhard Baumann and Yoshiaki Yasuno

1. Optical coherence tomography

Optical coherence tomography (OCT) is an optical imaging technique which was first introduced in the 90s [1]. OCT is based on low-coherence interferometry, where the internal microstructure of a tissue is revealed by measuring the backscattered and back-reflected light [2].

Over the past decades various implementations of OCT setups have been introduced. Figure 1 (a) shows a simplified time domain OCT (TD-OCT) setup based on a Michelson interferometer, in such setups mechanical scanning of the reference mirror is needed to ensure interference at different depth positions. Next, Fourier-domain (FD) OCT was introduced, which has a significant sensitivity advantage compared to TD-OCT [3]. FD-OCT systems can either be spectrometer or swept source (SS) based, see Fig. 1 (b). In spectrometer based OCT setups a spectrometer collects the interference signal of a broadband light source. In contrast to a SS-OCT setup in which, a narrowband light source sweeps through a broad spectral range and the interference signal is measured as a function of time using for example a photodetector [2].

In both, TD and FD-OCT a beam is emitted from the light-source to the reference and sample arm. In the sample arm the beam is backscatter and reflected form the internal structures of the tissue. The resulting interference pattern from sample and reference arm is measured by a detector. By post-processing this data a depth profile of the measured sample is gained. A single depth scan (z-direction) is called an A-scan. If the beam is scanned in lateral direction (x-direction), a B-scan or cross-sectional image is acquired. If the beam is also scanned in the other lateral direction (y-direction), a whole volume, or C-scan, is acquired [2]. Figure 1 (c) shows exemplary images of an OCT A-scan, B-scan and C-scan acquired in a one-month old wildtype zebrafish.

In OCT a low coherent light source is utilized which is characterized by a statistical phase discontinuity over a certain distance known as the coherence length. This coherence length is inversely proportional to the bandwidth of the light source and defines the axial resolution. A shorter coherence length results in a finer resolution. The axial resolution (Δ_z) of a state-of-the-art OCT systems is in the range of 1-15 μm [4] and can be estimated by:

$$\Delta_z = \frac{2\ln(2)}{\pi}\frac{\lambda^2}{\Delta\lambda}$$

Where λ is the central wavelength and Δλ is the bandwidth of the used light source. This formula shows, that the lower the central wavelength and the boarder the used bandwidth is, the lower the axial resolution can get [2].

The chosen wavelength region also defines the possible imaging range in depth. The lower the central wavelength the stronger is the scattering and absorption of light in biological tissue and this decreases the possible imaging depth. Therefore most conventional OCT setups are working in the near-infrared wavelength region where a depth range of a couple of millimeters can be achieved. Furthermore, there is tradeoff between lateral resolution and depth of field, i.e. using a high transverse resolution further decreases the depth range. The lateral resolution ($\Delta_{x,y}$) of an OCT setup is independent from the axial resolution and the same principle as in microscopy applies:

$$\Delta_{x,y} = \frac{4\lambda}{\pi}\frac{f}{D}$$

Where λ is the central wavelength, f is the focal length of the objective lens and D the entrance pupil [2].

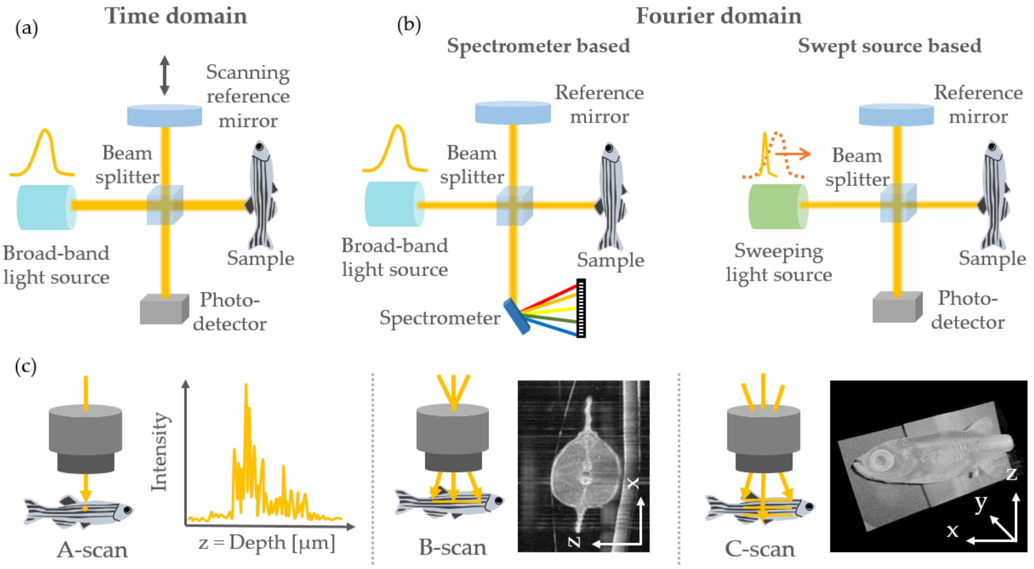

Figure. S1 Different types of optical coherence tomography (OCT) setups. (a) Time domain and (b) Fourier domain OCT system. (c) The difference between a depth-scan (A-scan), a cross-sectional image (B-scan) and a volume (C-scan) in OCT.

The acquisition time of an OCT scan is determined by the A-scan speed and the total amount of pixels utilized. Typical A-scan rates are in the range of multiple kHz and more recent even in the MHz range [5]. This results in volume acquisition times of a couple of seconds or less, making OCT a real-time imaging technique.

2. Comparison between selected OCT setups in zebrafish-based research

Table 1 gives an overview over various imaging parameters, the zebrafish development stage and area investigated in selected OCT publications.

Table S1. Comparison of system parameters of selected publications in the field of OCT-based zebrafish research. (Hours-post-fertilization (hpf), Days-post-fertilization (dpf), No information available (N/A), Jones-Matrix OCT (JM-OCT), Polarization-sensitive OCT (PS-OCT), Orthogonal-polarization-gating OCT (OPG-OCT), Swept-source OCT (SS-OCT), Spectral-domain OCT (SD-OCT), Time-domain OCT (TD-OCT)).

	Year	Fish age	Imaged area	OCT setup	Wavelength range	Axial resolution	Lateral resolution	A-scan rate
Lichtenegger A. et al.[6]	2022	1-month	Tail	JM-OCT	1310.0 nm	14.0 µm	18.1 µm	50 kHz
Yang D. et al.[7]	2022	6-months	Brain/Skull	PS-OCT	840.0 nm	3.4 µm	8.0 µm	25 kHz
Li K. et al.[8]	2022	72 hpf	Yolk sac	OPG-OCT	1310.0 nm	8.9 µm	18.0 µm	76 kHz
Alam Z. et al.[9]	2022	3-6 dpf	Whole fish	SS-OCT	1060.0 nm	4.5 µm	N/A	100 kHz
Quint W.H. et al.[10]	2022	2-6 months	Eye	SD-OCT	900.0 nm	2.0 µm	4.0 µm	80 kHz
Zhang J. et al.[11]	2022	90 dpf	Skull	SD-OCT	830.0 nm	15.2 µm	12.7 µm	18kHz
Huckenpahler et al.[12]	2020	12-36 months	Eye	SD-OCT	878.4 nm	N/A	N/A	N/A
Haindl R. et al.[13]	2020	96-120 hpf	Whole fish	SD-OCT	780.0 nm	2.4 µm	2.4 µm	10 kHz
Bozic I. et al.[14]	2018	>3 months	Eye	SD-OCT	855.0 nm	2.6 µm	2.8 µm	125 kHz
Chen Y. et al.[15]	2016	2-5 dpf	Tail	SD-OCT	825.0 nm	2.8 µm	2.8 µm	NA
Zhang J. et al.[16]	2015	Adult	Brain	SD-OCT	1325 nm	12.0 µm	13.0 µm	76 kHz
Cui D. et al.[17]	2014	3 dpf	Fin	SD-OCT	930.0 nm	1.27 µm	1.7 µm	10 kHz
Kagemann L. et al.[18]	2008	24-120 hpf	Whole fish	SD-OCT	840.0 nm	3.5 µm	N/A	25 kHz
Boppart S.A. et al.[19]	1996	1-24 hpf	Whole fish	TD-OCT	1300 nm	12.0 µm	30.0 µm	N/A

References.

1. Huang, D.; Swanson, E.A.; Lin, C.P.; Schuman, J.S; Stinson, W.G.; Chang, W.; Hee, M.R.; Flotte, T.; Gregory, K.; Puliafito, C.A.; et al. Optical coherence tomography. *Science* **1991**, *254*, 1178–1181.
2. Drexler, W.; Fujimoto, J. G.; *Optical Coherence Tomography Technology and Applications*. Springer Science & Business Media Springer: Heidelberg, German, 2015; pp 3-65.
3. Leitgeb, R.;Hitzenberger, C.; Fercher, A.; Performance of fourier domain vs time domain optical coherence tomography. *Opt. Express* **2003**, *11*, 889.
4. Popescu, D. P.;Choo-Smith, L. P. in.;Flueraru, C.;Mao, Y.;Chang, S.;Disano, J.;Sherif, S.; Sowa, M. G.; Optical coherence tomography: Fundamental principles, instrumental designs and biomedical applications. *Biophys. Rev.* **2011**, *3*, 155–169.
5. Klein, T.;Neubauer, A.;Huber, R.;Wieser, W.;Reznicek, L.; Kampik, A.; Multi-MHz retinal OCT. *Biomed. Opt. Express* **2013**, *4*, 1890–1908.
6. Lichtenegger, A.; Tamaoki, J.; Licandro, R.; Mori, T.; Mukherjee, P.; Bian, L.; Greutter, L.; Makita, S.; Wöhrer, A.; Matsusaka, S.; et al. Longitudinal investigation of a xenograft tumor zebrafish model using polarization-sensitive optical coherence tomography. *Sci. Rep. 2022 121* **2022**, *12*, 1–13.
7. Yang, D.;Yuan, Z.;Hu, M.; Liang, Y.; Zebrafish brain and skull imaging based on polarization-sensitive optical coherence tomography. *J. Biophotonics* 2022, e202200112. https://doi.org/10.1002/jbio.202200112.
8. Li, K.;Wang, Y.;Liu, Y.;Li, W.;Weng, Z.;Li, H.;He, Y.; Li, Z.; Morphological characteristics of zebrafish's yolk sac for malformation based on orthogonal-polarization-gating optical coherence tomography. *J. Biophotonics* 2022, e202200098. https://doi.org/10.1002/jbio.202200098
9. Alam, Z.; Poddar, R.; An in-vivo depth-resolved imaging of developing zebrafish microstructure and microvasculature using swept-source optical coherence tomography angiography. *Opt. Lasers Eng.* **2022**, *156*, 107087.
10. Quint, W. H.;Tadema, K. C. D.;Crins, J. H. C.;Kokke, N. C. C. J.;Meester-Smoor, M. A.;Willemsen, R.;Klaver, C. C. W.; Iglesias, A. I.; Zebrafish: An In Vivo Screening Model to Study Ocular Phenotypes. *Transl. Vis. Sci. Technol.* **2022**, *11*, 17–17.
11. Xiang, X.; Gao, W.; Xu, Y.; Zhang, Y.; Lu, T.; Gan, S.; Huang, J.; Li, Z.; Huang, L.; Liao, Y.; et al.; Study on promoting regeneration of zebrafish skull by phycocyanin characterized by in vivo optical coherence tomography. *J. Biophotonics* **2022**, *15*, e202100333.
12. Huckenpahler, A. L.;Lookfong, N. A.;Warr, E.; Carroll, E. H.; Collery, R. F.; Noninvasive imaging of cone ablation and regeneration in zebrafish. *Transl. Vis. Sci. Technol.* **2020**, *9*, 1–14.
13. Haindl, R.; Deloria, A.J.; Sturtzel, C.; Sattmann, H.; Rohringer, W.; Fischer, B.; Andreana, M.; Unterhuber, A.; Schwerte, T.; Distel, M.; et al.; Functional optical coherence tomography and photoacoustic microscopy imaging for zebrafish larvae. *Biomed. Opt. Express* **2020**, *11*, 2137–2151.
14. Bozic, I.;Li, X.; Tao, Y.; Quantitative biometry of zebrafish retinal vasculature using optical coherence tomographic angiography. *Biomed. Opt. Express* **2018**, *9*, 1244–1255.
15. Chen, Y.;Trinh, L. A.;Fingler, J.; Fraser, S. E.; Phase variance optical coherence microscopy for label-free imaging of the developing vasculature in zebrafish embryos. *J. Biomed. Opt.* **2016**, *21*, 126022.
16. Zhang, J.;Ge, W.; Yuan, Z.; In vivo three-dimensional characterization of the adult zebrafish brain using a 1325 nm spectral-domain optical coherence tomography system with the 27 frame/s video rate. *Biomed. Opt. Express* **2015**, *6*, 3932–3940.
17. Cui, D.;Liu, X.;Zhang, J.;Yu, X.;Ding, S.;Luo, Y.;Gu, J.;Shum, P.; Liu, L.; Dual spectrometer system with spectral compounding for 1-μm optical coherence tomography in vivo. *Opt. Lett.* **2014**, *39*, 6727–6730.
18. Kagemann, L.; Ishikawa, H.; Zou, J.; Charukamnoetkanok, P.; Wollstein, G.; Townsend, K.A.; Gabriele, M.L.; Bahary, N.; Wei, X.; Fujimoto, J.G.; et al.; Repeated, noninvasive, high resolution spectral domain optical coherence tomography imaging of zebrafish embryos. *Mol. Vis.* **2008**, *14*, 2157–70.
19. Boppart, S. A.;Brezinski, M. E.;Bouma, B. E.;Tearney, G. J.; Fujimoto, J. G.; Investigation of developing embryonic morphology using optical coherence tomography. *Dev. Biol.* **1996**, *177*, 54–63.
